# Supplementary material for: Extended 2,2′-Bipyrroles: New Monomers for Conjugated Polymers with Tailored Processability
Source: Polymers (Basel). 2019 Jun 20;11(6):1068. doi: 10.3390/polym11061068 (PMC6630584; doi:10.3390/polym11061068)
Supplement: Supplementary file 1 [file polymers-11-01068-s001.pdf]

Extended 2,2′-bipyrroles: new monomers for conjugated polymers with tailored processability

Robert Texidó[1], Gonzalo Anguera[1], Sergi Colominas[3], Salvador Borrós[1,2] and David Sánchez-García [1,*]

[1] Grup d'Enginyeria de Materials (GEMAT), Institut Químic de Sarrià, Universitat Ramon Llull, Via Augusta, 390, 08017 Barcelona, Spain; roberttexido@gmail.com (R.T.); gonzaloanguerap@iqs.url.edu (G.A.); salvador.borros@iqs.url.edu (S.B.G.)

[2] Centro de Investigación Biomédica en Red en Bioingeniería, Biomateriales y Nanomedicina (CIBER-BBN), Zaragoza 50018, Spain

[3] Electrochemical Methods Laboratory - Analytical and Applied Chemistry Department at Institut Químic de Sarrià, Universitat Ramon Llull, Via Augusta, 390, 08017 Barcelona, Spain; sergi.colominas@iqs.url.edu (S.C)

Table of Contents

I. Synthesis 2

II. NMR spectra 5

III. Characterization of the polymers prepared by chemical oxidation 6

IV. Absorption and Fluorescence spectra 8

V. First cyclic voltammetry cycle vs Fc 9

VI. MALDI – TOF 10

VII. Chemical Structures of the polymers 10

I. Synthesis

Diethyl 5,5'-dibromo-4,4'-diphenyl-1*H*,1'*H*-[2,2'-bipyrrole]-3,3'-dicarboxylate (0.34 mmol) and 1-methyl-2-(trimethylstannyl)-1*H*-pyrrole (0.85 mmol) were dissolved in 20 ml of dry toluene. Then the solution was flushed with N_2 20 min, $Pd(PPh_3)_4$ (0.12 g, 0.1 mmol) was added and flushed 5 min more. The mixture was stirred for 3 hours at 140 ºC in a microwave instrument. The mixture was filtered to separate the precipitated catalyst and the solvent was removed under reduced pressure to obtain a brown oil. The product was obtained washing the brown oil with mixtures of AcOEt and Cy.

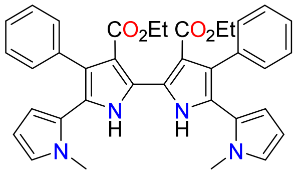

1c

Fig. S1. Chemical structure of monomer **1c**.

1c (98%, yellow powder) IR (KBr pellets)/cm^{-1}: 3432, 2982, 1654, 1444, 1410, 1177, 1021, 838, 723, 704. ^{1}H NMR (400 MHz, $CDCl_3$) δ: 13.36 (s, 2H), 7.27 – 7.16 (m, 10H), 6.55 (dd, *J* = 2.7, 1.8 Hz, 2H), 6.14 (dd, *J* = 3.7, 1.8 Hz, 2H), 6.09 (dd, *J* = 3.7, 2.7 Hz, 2H), 4.03 (q, *J* = 7.1 Hz, 4H), 3.24 (s, 6H), 0.84 (t, *J* = 7.1 Hz, 6H). ^{13}C NMR (100.6 MHz, $CDCl_3$) δ: 168.60, 136.78, 130.51, 128.49, 127.54, 126.93, 126.19, 124.59, 123.10, 122.71, 111.17, 111.13, 107.88, 60.84, 34.72, 13.46, 0.15. Anal. $C_{36}H_{34}N_4O_4$ Calculated: C73.70 H 5.84 N9.55, Found: C73.68 H5.97 N9.51. UV-Vis λ_{max}/nm (ε/$M^{-1}\cdot cm^{-1}$) $CHCl_3$: 383 ($1.6 \cdot 10^4$). m.p.: decomp. 245 ºC.

General methodology for obtaining polymers by chemical coupling (P-1a-c)

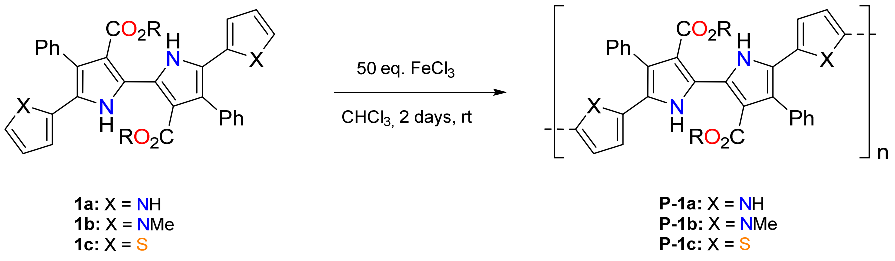

Fig. S2. Chemical oxidative coupling of monomers **1a-c** and chemical structure of polymers **P-1a-c**.

1a-c (1 eq.) was dissolved in 35 ml of dry chloroform and anhydrous $FeCl_3$ (50 eq.) was added in portions, then the solution was stirred for 2 days at room temperature under nitrogen. The solvent was removed under reduced pressure and the obtained solid was suspended in methanol and refluxed 20 min. The mixture was filtrated and solid washed with methanol to eliminate the $FeCl_3$.

The obtained solid was refluxed with THF and filtered to remove the insoluble part of the polymer. The filtrate was concentrated and the product was precipitated with the addition of methanol. Precipitate was cooled at 5 °C overnight and filtered to obtain a powder.

Table S1: properties of weight, absorbance and fluorescence of polymers prepared by chemical oxidation.

Monomer	M_w[a]	M_n[a]	PDI[a]	Absorbance[b]	Emission[c]	Quantum Yield[d]
**1a**	2300	1100	2.18	369	512	<1%
**1b**	2600	1100	2.46	408	499	0.04
**1c**	2300	1000	2.21	457	507	0.12

[a] Estimated from GPC (eluent THF, polystyrene standards). [b] All spectra were recorded in THF at a concentration of 0.1 mg/ml. [c] Emission spectra were measured with excitation at the maximum absorption of each polymer. [d] Quantum yields were determined in THF using a solution of quinine in 0.05M H_2SO_4 ($\Phi_F = 0.546$) as fluorescence standard.

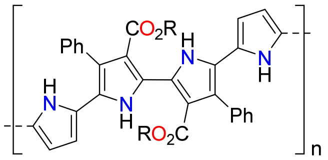

Fig. S3. Chemical structure of polymer **P-1a** (R=Et).

1a (5%) IR (KBr pellet)/cm^{-1}: 3413, 2926, 1719, 1548, 1465, 1384, 1269, 1184, 1093, 1022, 968, 843, 795, 771, 700. UV-VIS: λ_{max} = 369 nm, absorption up to 600 nm.

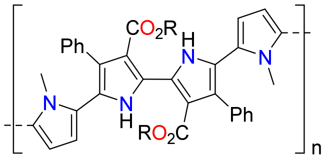

Fig. S4. Chemical structure of polymer **P-1b** (R=Et).

1b (21%) IR (KBr pellet)/cm^{-1}: 3433, 2922, 1669, 1614, 1448, 1419, 1174, 1020, 863, 756, 695. ^{1}H-NMR (400 Mhz, d_6-DMSO) δ: 12.53 (brs, 1H), 9.20 – 8.98 (m, 2H), 7.64 – 7.36 (m, 6H), 4.34 – 3.86 (m, 10H), 1.18 (m, 6H). UV-VIS: λ_{max} = 408 nm, absorption up to 600 nm.

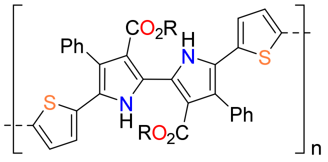

Fig. S5. Chemical structure of polymer **P-1c** (R=Et).

1c (17%) IR (KBr pellet)/cm^{-1}: 3432, 2924, 1658, 1418, 1181, 1063, 930, 794, 770, 699. ^{1}H-NMR (400 Mhz, d_6-DMSO) δ: 12.31 (m, 2H), 7.49 – 7.35 (m, 6H), 7.30 – 7.15 (m, 6H), 6.97 (m, 2H), 3.83 (m, 4H), 0.83 (m, 6H). UV-VIS: λ_{max} = 457 nm, absorption up to 515 nm.

II. NMR spectra

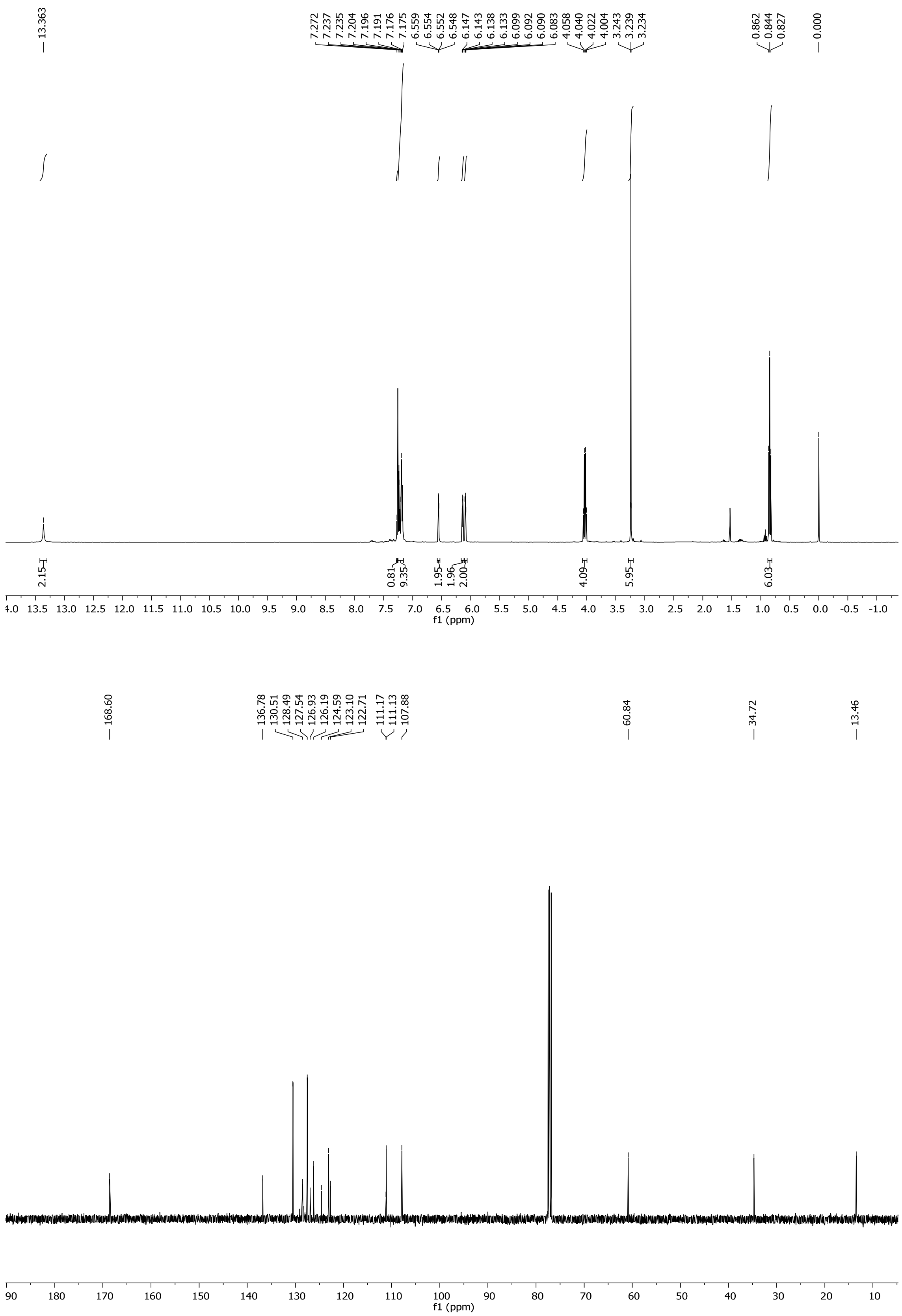

Fig. S6. ^{1}H-NMR (a) and ^{13}C-NMR (b) spectra of **1c** in $CDCl_3$.

III. Characterization of the polymers prepared by chemical oxidation

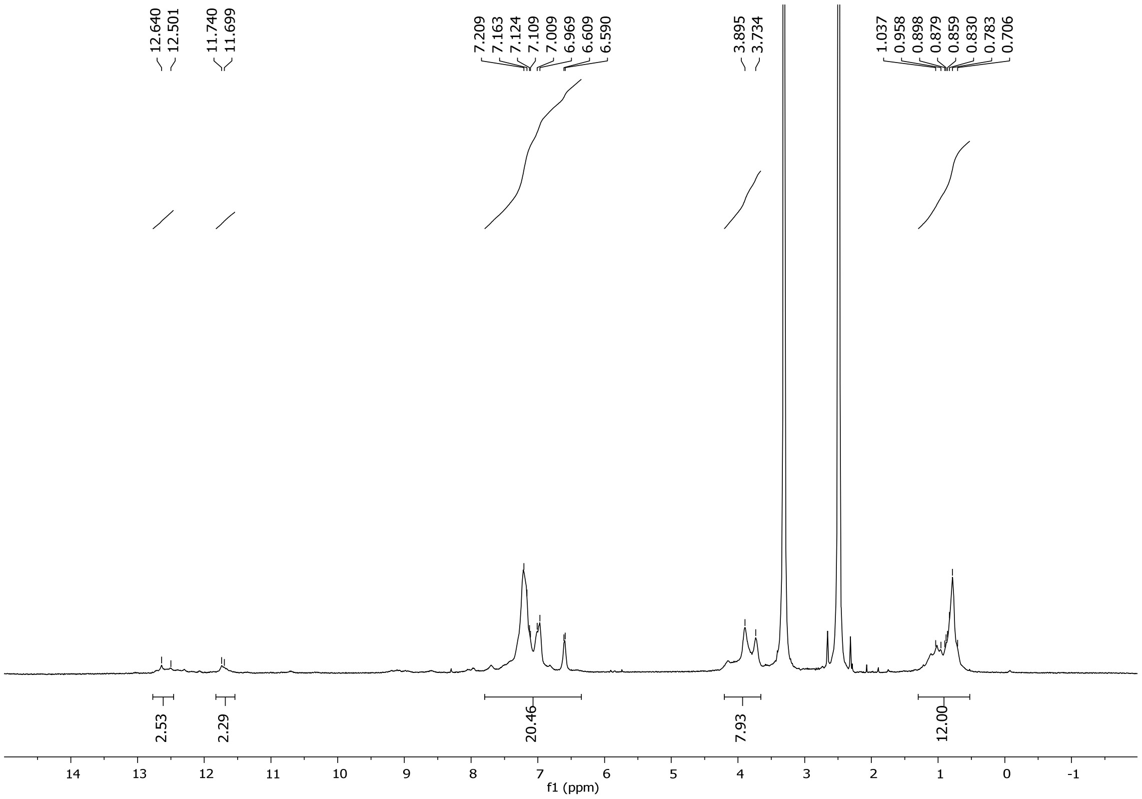

Fig. S7. ^{1}H-NMR spectrum of **P-1a** in DMSO-d_6.

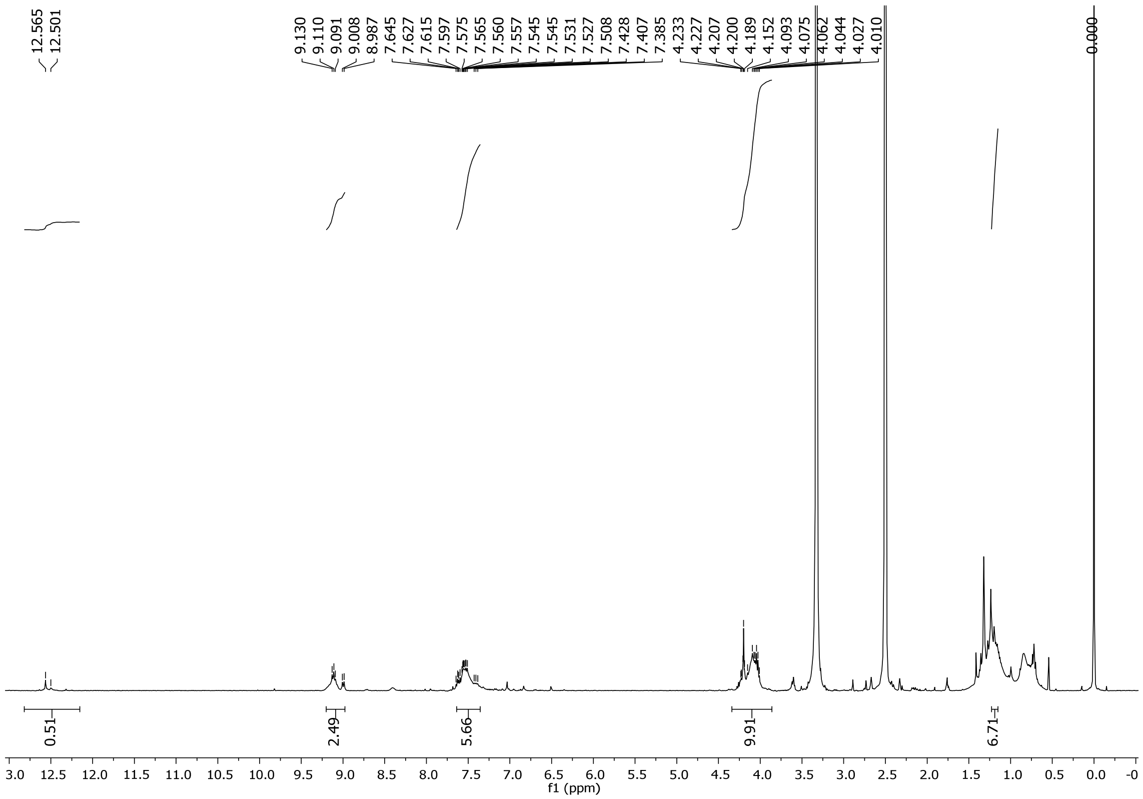

Fig. S8. ^{1}H-NMR spectrum of **P-1b** in DMSO-d_6.

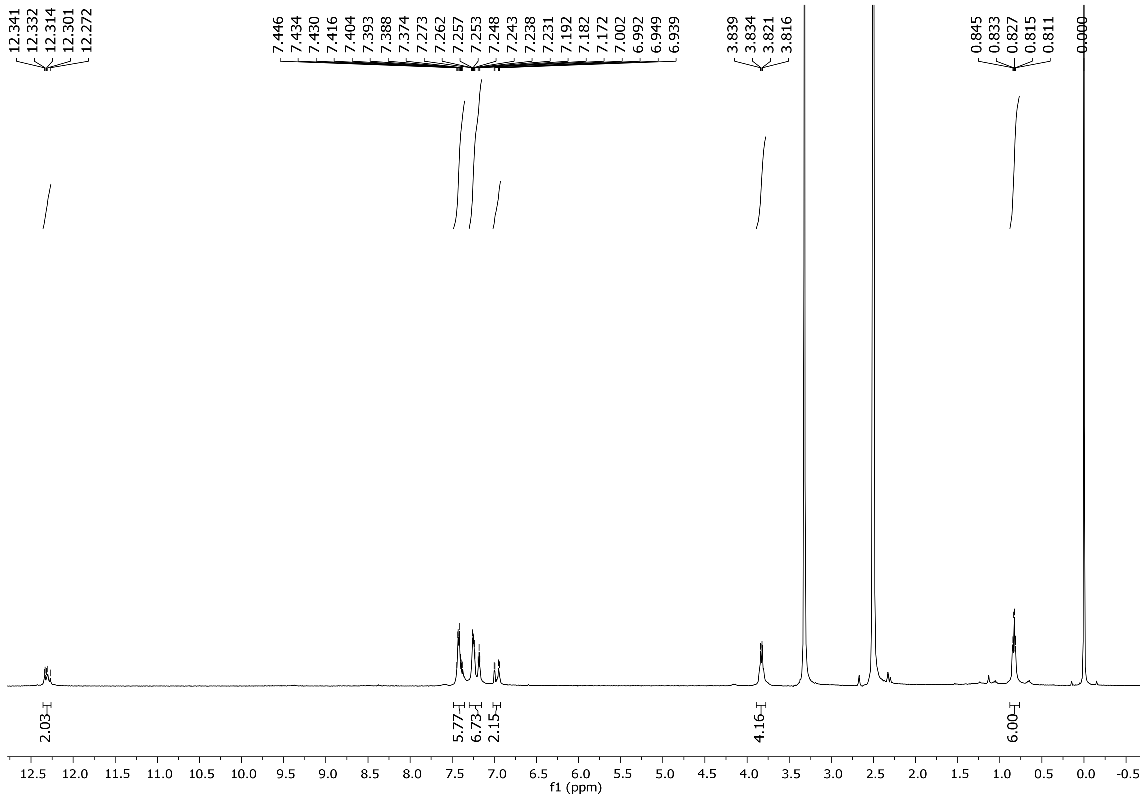

Fig. S9. ^{1}H-NMR spectrum of **P-1c** in DMSO-d_6.

IV. Absorption and Fluorescence spectra

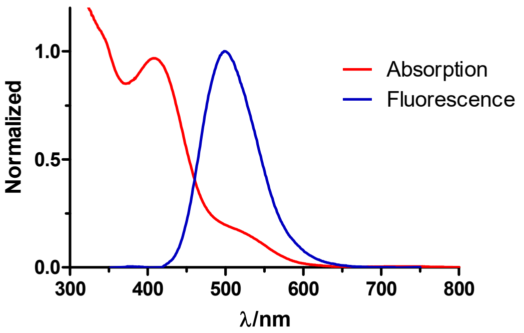

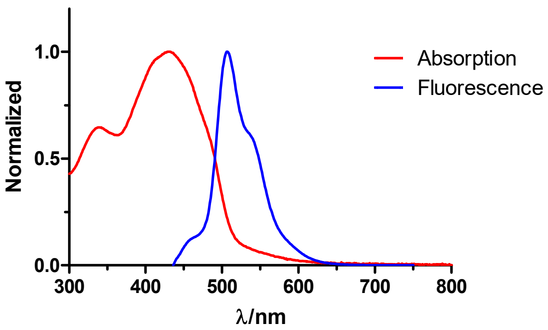

Fig. S10. Absorption and Fluorescence spectra normalized for polymers **P-1b** and **P-1c** in THF.

V. First cyclic voltammetry cycle vs Fc

1a

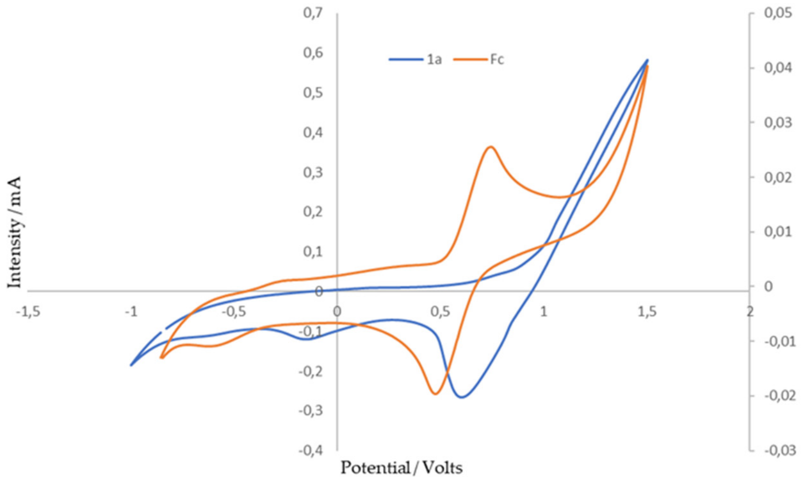

1b

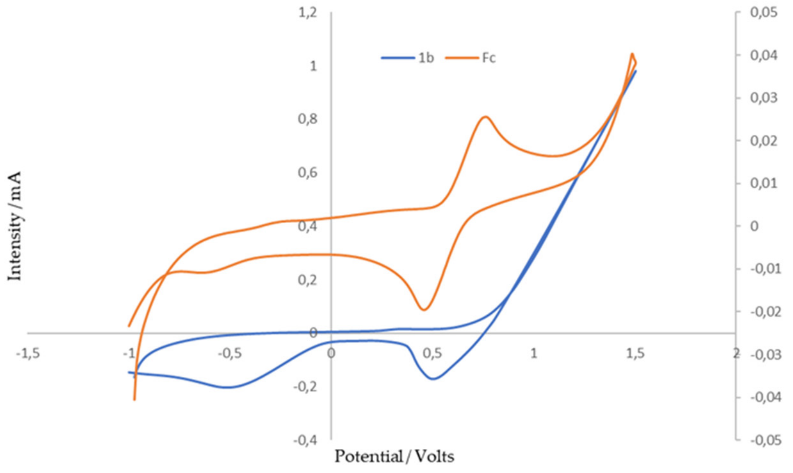

1c

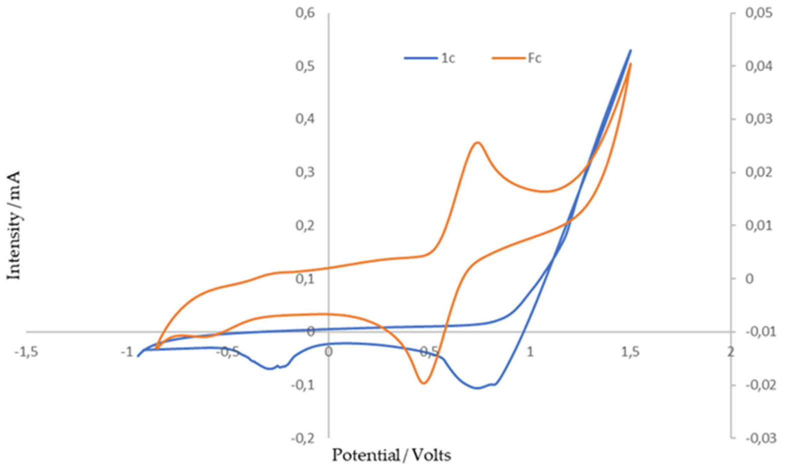

Fig. S11. First cyclic voltammetry of **1a-c** 2,2'-bipyrrole monomers overlapped with Ferrocene/Ferrocenium couple.

VI. MALDI – TOF

Table S2: possible fragmentation peaks

m/z	1a
**558.2**	Monomer
**814.2**	Monomer + Tetra butyl ammonium
**919.0**	Monomer + Tetra butyl ammonium perchlorate
**1116.4**	Dimer
**1370.7**	Dimer + Tetra butyl ammonium
**1684.7**	Trimer
**1927.9**	Trimer + Tetra butyl ammonium
**2227.9**	Oligomer n = 4
**2786.1**	Oligomer n = 5

m/z	1b
**586.2**	Monomer
**656.3**	Monomer + Perchlorate - Ethyl group
**707.1**	Monomer + Perchlorate -2 Ethyl groups
**810.3**	Monomer + Tetra butyl ammonium
**847.3**	Monomer + 3Perclorate groups - Esther group
**1012.4**	Dimer - 2 Phenyl groups - 2 Esther groups
**1091.4**	Dimer - Phenyl group
**1170.5**	Dimer
**1276.6**	Dimer + Perchlorate

m/z	1c
**592.2**	Monomer
**672.0**	Monomer + Perchlorate
**810.3**	Monomer + Tetra butyl ammonium
**972.2**	Monomer + Tetra butyl ammonium perchlorate - Ethyl group
**1182.3**	Dimer
**1285.3**	Dimer + Perchlorate
**1443.4**	Dimer + Tetra butyl ammonium

VII. Chemical Structures of the polymers

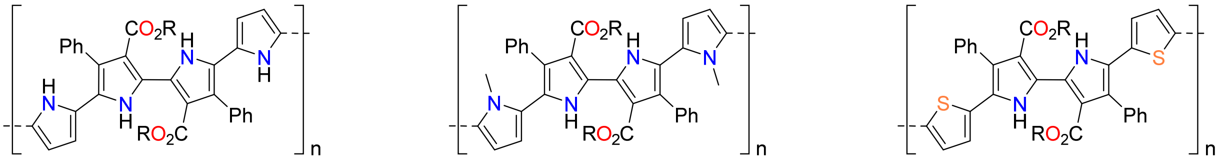

Fig. S12. Chemical structures of 2,2'-bipyrrole based polymers **P-1a-c** (R=Et).
